# Success rates and adherence to antiretroviral therapy among treatment-naïve patients in Davao City, Philippines: A ten-year retrospective cohort study

**DOI:** 10.1016/j.dialog.2024.100195

**Published:** 2024-09-24

**Authors:** Reigner Jay B. Escartin, Rvin John T. Servillon, Jesille Mae A. Legarta, Stephanie Kate G. Arriola, Princess Faiqah A. Brahim, Dennis Louis M. Braza, Aireen V. Salva, Alfredo A. Hinay

**Affiliations:** a College of Medical and Biological Sciences, University of the Immaculate Conception, Davao City, Philippines; b Graduate School Department, University of the Immaculate Conception, Davao City, Philippines

**Keywords:** HIV treatment success, HIV treatment adherence, Treatment-Naïve patients

## Abstract

**Background:**

Antiretroviral therapy (ART) has significantly improved the prognosis and quality of life of HIV/AIDS patients. However, ART success is greatly influenced by patient adherence to the treatment regimens. This study aimed to assess the association between patient adherence to ART and the treatment success rate among antiretroviral-naïve patients in Davao City, Philippines.

**Methods:**

This study utilized a 10-year retrospective cohort design, including 517 antiretroviral-naïve patients from an HIV treatment hub in Davao City, the Philippines. Using strict inclusion criteria, 183 antiretroviral-naïve patients were analyzed.

**Findings:**

The study found significant associations between the type of ART (*p* *=* 0.011) and the timing of ART initiation (*p* = 0.006) with both patient adherence and the ART success rate. Specifically, patients who achieved sustained viral suppression were predominantly those who initiated ART early, with 71.6 % (*n* = 131) of them prescribed a regimen consisting of 2 NRTIs and 1 NNRTI. Moreover, 73.8 % of patients demonstrated good adherence (<50 HIV copies/mL). Importantly, patient adherence to ART was strongly correlated with treatment success rate.

**Interpretation:**

This study highlights the significance of adherence to antiretroviral therapy (ART) for successful treatment outcomes among antiretroviral-naïve patients living with HIV/AIDS. Early initiation of ART and consistent adherence to treatment regimens are essential for achieving sustained viral suppression and improving treatment effectiveness.

## 1 Introduction

Acquired immunodeficiency syndrome (AIDS) is the worst pandemic known in the history of humankind [1]. It is the world's sixth leading cause of death, a fact that has not changed since 2000 [2]. Globally, approximately 38 million people were living with Human Immunodeficiency Virus (PLHIV or PLWH) at the end of 2019, and 81 % of these people knew their status; however, only 67 % (25.4 million) had access to Antiretroviral Therapy (ART). This may suggest that not everyone receives antiretroviral therapy [3] or that some individuals discontinue receiving antiretroviral treatments [4]. As a result, 690,000 people died of AIDS in 2019 [3]. In the Philippines, 196 of 1249 newly confirmed HIV-positive individuals developed AIDS-defining symptoms at the time of diagnosis in 2019 [5]. This problem is not new, and may be due to a strong relationship between the level of adherence of patients to ART and the risk of HIV progression to AIDS [6]. Locally, the Davao Region ranked 7th, with the highest number of newly diagnosed individuals with HIV/AIDS in the Philippines [7].

ART is the best treatment method for preventing the progression of HIV to AIDS [8,9]; however, its effectiveness depends on the extent of patient adherence to ART [10]. According to Gunthard et al., optimal adherence to ART significantly contributes to high treatment success rates among HIV-infected individuals [11]. Hence, at least 95 % adherence is required to achieve a 90 % success rate for antiretroviral treatments.

Although there has been a reduced number of newly infected persons and increased ART coverage in previous years, efforts to achieve and maintain optimal adherence are still lacking. In addition, despite the widely accessible effective treatment options for AIDS such as nucleoside reverse transcriptase inhibitors (NRTIs), non-nucleoside reverse transcriptase inhibitors (NNRTIs), and protease inhibitors (PIs), cases of HIV infection keep on progressing to AIDS [12,13]. In addition, in resource-rich countries, antiretroviral effectiveness has already been demonstrated; however, only a few studies have been conducted in resource-limited countries that state ART effectiveness [14].

Consequently, this study was conducted to investigate the association between the ART success rate and adherence of antiretroviral-naïve patients to an HIV Treatment Hub in Davao City, Philippines. The findings of this study are crucial for both healthcare providers and policymakers, as they provide valuable insights into the effectiveness of ART and the factors influencing treatment adherence among HIV-positive individuals in Davao City, the Philippines. By improving treatment adherence and success rates, this study has the potential to positively impact public health outcomes and reduce the burden of HIV/AIDS in this region.

## 2 Methods

### 2.1 Study design and setting

This retrospective cohort study was conducted at the Reproductive Health and Wellness Center (RHWC) located on Jacinto Street in Davao City, the Philippines. RHWC serves as the primary HIV care facility for Region XI, which has an estimated population of approximately 5.8 million. It provides crucial healthcare services for individuals living with HIV/AIDS.

### 2.2 Population

All adult (≥15 years) treatment-naïve patients who started antiretroviral therapy between 2012 and 2021 were included.

### 2.3 Eligibility criteria

1. Treatment-naïve patients aged ≥15 years who attended the clinic between 2012 and 2021.
2. Patients who initiated antiretroviral therapy (ART) during the specified period and had baseline viral load (VL) measurements and CD4 cell count data before starting ART.
3. Patients with follow-up data including at least one CD4 cell count and one plasma HIV-1 RNA measurement (VL) after ART initiation.
4. Patients with documented AIDS-defining conditions.
5. Patients for whom information on the type of combination antiretroviral therapy (cART) was available.
6. Patients with pharmacy records indicated the following.
  a. Number of pills dispensed per month.
  b. Number of pills taken per day.

### 2.4 Sampling procedure

A total of 517 treatment-naïve patients records were available from the administrative databases and medical records of the Reproductive Health and Wellness Center (RHWC), Davao City, Philippines. Each record underwent a thorough screening process to determine eligibility based on predefined inclusion and exclusion criteria. After screening, 183 patients met the inclusion criteria and were considered for further analysis.

### 2.5 Research procedure

Data completeness and consistency were checked using Microsoft Excel 2013. The data were extracted by a trained medical technologist. The data extraction format included sociodemographic characteristics (age and sex), type of ART, timing of ART initiation, number of pills dispensed per month, number of pills taken per day, laboratory markers (baseline CD4 cell count, viral load, and AIDS-defining condition before regimen initiation), and follow-up records (at least one CD4 cell count and one plasma HIV-1 RNA measurement (VL) during follow-up after starting ART, and availability of AIDS-defining condition records during follow-up after starting ART). Data collection and extraction processes were supervised by the investigators. All completed data were examined for clarity and consistency before analysis.

### 2.6 Data analysis

The Data were analyzed using the IBM Statistics Software. Data were analyzed using multivariate logistic regression to determine the linear relationship between a series of predictor variables (e.g., patient characteristics) and outcome variables (e.g., patient adherence and ART success) and to examine those that best predicted a certain outcome. The Eta coefficient test was used to analyze and measure the strength of a nonlinear or curvilinear association, which is the relationship between a categorical variable (e.g., virologic response) and scale variable (e.g., timing of ART initiation). Univariate logistic regression was used to analyze the association between patient adherence to ART and ART success.

## 3 Results

### 3.1 Patient characteristics of antiretroviral-Naïve patients

The majority of antiretroviral-naive patients were male (98.4 %, *n* = 180) and 75.4 % (*n* = 138) were younger than 30 years of age. Most patients (39.3 %, *n* = 72) initiated ART within seven days after the confirmed HIV diagnosis, while 31.7 % (*n* = 58) experienced a delay in initiating ART. Additionally, 94 % (*n* = 132) of patients began combination antiretroviral therapy (cART) with two NRTIs plus one NNRTI, while less than 3.8 % received different ART combinations. Regarding the number of pills dispensed per month, the majority of patients (78.1 %, *n* = 143) received 30 pills, and typically, one pill was taken per day (97.3 %, *n* = 178).

### 3.2 Patient adherence to antiretroviral therapy based on biological marker

Table 2 shows the adherence of the patients based on whether they have successfully suppressed the viral load to undetectable levels (good adherence <50 copies/mL) or have failed to suppress it (poor adherence >50 copies/mL). Most patients (73.8 %, *n* = 135) successfully suppressed viral load, whereas 26.2 % (*n* = 48) failed. This means that these patients are at a considerable risk of passing HIV to others. (See Table 1.)

**Table 2:** Patient adherence to antiretroviral therapy based on biological marker (Viral Suppression after taking ART).

		n	%
Adherence	Good adherence	135	73.8
	Poor adherence	48	26.2

**Table 3:** Clinical parameters of the success rate of antiretroviral therapy (ART).

		Baseline		Follow-up	
		n	%	n	%
CD4 Count	>500 cells/	26	14.2	13	7.1
	200–499 cells/	57	31.1	20	10.9
	<200 cells/	59	32.2	5	2.7
	No data	41	22.4	145	79.2
Viral load	<50 copies/mL	73	39.9	134	73.2
	>50 copies/mL	110	60.1	49	26.8
AIDS-defining condition	Stage 1: No AIDS-defining condition	26	14.2	13	7.1
	Stage 2: No AIDS-defining condition	57	31.1	20	10.9
	Stage 3: With documented AIDS-defining condition	59	32.2	5	2.7
	Stage Unknown: No information on presence of AIDs-defining condition	41	22.4	145	79.2

**Table 4:** Success rate of antiretroviral therapy (ART) based on viral load suppression after taking ART.

		n	%
Success	Improved (sustained viral suppression <50 copies/mL)	134	73.2
	No improvement (unsustained viral suppression >50 copies/mL)	49	26.8

**Table 5:** Association between patient characteristics and patient adherence to antiretroviral therapy (ART).

		Adherence						
		Good		Poor				
		n	%	n	%	*X*^*2*^	*p*	Description
Age	<30 years	99	54.1	39	21.3	1.861	0.602	Not significant
	31–40 years	33	18.0	9	4.9			
	41–50 years	2	1.1	0	0.0			
	>50 years	1	0.5	0	0.0			
Sex	Male	133	72.7	47	25.7	0.080	0.778	Not significant
	Female	2	1.1	1	0.5			
ART Drug	2 NRTIs + NNRTIs	131	71.6	41	22.4	9.067	0.011	Significant
	2 NRTIs +1 INSTIs	2	1.1	5	2.7			
	2 NRTIs + PK Enhancer	2	1.1	2	1.1			
Timing of ART Initiation	Early ART (<7 days of confirmed HIV diagnosis)	62	33.9	10	5.5	7.657	0.006	Significant
	Delayed ART (>7 days of confirmed HIV diagnosis)	38	20.8	20	11			
No. of Pills Dispensed	30 pills dispensed	103	56.3	40	21.9	1.462	0.481	Not significant
	60 pills dispensed	30	16.4	8	9.0			
	90 pills dispensed	2	1.1	0	0.0			
No. of Pills Taken	1	133	72.7	45	24.6	3.202	0.202	Not significant
	2	1	0.5	2	1.1			
	3	0	0.0	0	0.0			
	4	1	0.5	1	0.5

**Table 6:** Association between patient characteristics and antiretroviral therapy success.

		Success						
		Improved		Not improved				
		n	%	n	%	*X*^*2*^	*p*	Description
Age	<30 years	98	53.6	40	21.9	2.053	0.067	Not Significant
	31–40 years	33	18.0	9	4.9			
	41–50 years	2	1.1	0	0.0			
	>50 years	1	0.5	0	0.0			
Sex	Male	132	72.1	48	26.2	0.067	0.796	Not Significant
	Female	2	1.1	1	0.5			
ART Drug	2 NRTIs + NNRTIs	130	71.0	42	23.0	8.706	0.013	Significant
	2 NRTIs +1 INSTIs	2	1.1	5	2.7			
	2 NRTIs + PK Enhancer	2	1.1	2	1.1			
Date of ART started	Early ART (<7 days of confirmed HIV)	60	46.2	12	9.2	5.495	0.019	Significant
	Delayed ART (>7 days of confirmed HIV)	38	29.2	20	15.4			
No. of Pills Dispensed	30 pills dispensed	101	55.2	42	23.0	2.575	0.276	Not significant
	60 pills dispensed	31	17.0	7	3.8			
	90 pills dispensed	2	1.1	0	0.0			
No. of Pills Taken	1	132	72.1	46	25.1	3.064	0.216	Not significant
	2	1	0.5	2	1.1			
	3	0	0.0	0	0.0			
	4	1	0.5	1	0.5

**Table 7:** Strength of Association between timing of ART Initiation and Viral Suppression after taking ART.

		Eta (η)	Eta squared ($\eta^{2}$)	Interpretation
Eta	Virologic Response Independent	0.683	0.466	Strong Effect
	Date interval from the date of confirmed HIV diagnosis to date ART initiation Dependent	0.139		

Eta squared interpretation: 0.01, small effect; 0.06, moderate effect;0.14, strong effect.

**Table 8:** Association between patient adherence and success rate of antiretroviral therapy (ART).

		Success						
		Improved		No improvement				
		n	%	n	%	*x*^*2*^	p	Description
Adherence	Good adherence	133	72.7	2	1.1	167.956	*p < 0.001*	Significant
	Poor adherence	1	0.5	47	25.7

**Table 1:** Patient characteristics of antiretroviral-naive patients.

		n	%
Age	<30 years	138	75.4
	31–40 years	42	23.0
	41–50 years	2	1.1
	>50 years	1	0.5
Sex	Male	180	98.4
	Female	3	1.6
Anti-Retroviral Drug	2 NRTIs + NNRTIs	172	94.0
	2 NRTIs +1 INSTIs	7	3.8
	2 NRTIs + PK Enhancer	4	2.2
Timing of ART initiation	Early ART (<7 days of confirmed HIV diagnosis)	72	55.4
	Delayed ART (>7 days of confirmed HIV diagnosis)	58	44.6
Number of pills dispensed	30 pills dispensed	143	78.1
	60 pills dispensed	38	20.8
	90 pills dispensed	2	1.1
Number of Pills Taken	1	178	97.3
	2	3	1.6
	3	0	0.0
	4	2	1.1

### 3.3 Clinical parameters of the success rate of antiretroviral therapy (ART)

The overall clinical responses of antiretroviral-naïve patients at baseline and during follow-up after taking antiretroviral drugs are presented in Table 3. Before the initiation of ART, 60.1 % (*n* = 110) of the patients had >50 copies/mL and 32.2 % (*n* = 59) had AIDS-defining conditions (<200 CD4 cells/mm^3^). After taking antiretroviral drugs, 73.2 % (*n* = 134) of patients successfully suppressed the viral load to <50 copies/mL.

### 3.4 Success rate of antiretroviral therapy (ART) based on viral load suppression after taking ART

Table 4 shows the success rate of ART based on viral suppression in patients administered antiretroviral drugs. It is evident that 73.2 % (*n* = 134) of the patients had sustained viral suppression, maintaining viral load at undetectable levels (<50 copies/mL), while 26.8 % (*n* = 49) of the patients showed no improvement after ART initiation. This means that individuals with detectable levels of HIV (>50 copies/mL), even after taking ART, have a considerable risk of passing HIV to others.

### 3.5 Association between patient characteristics and patient adherence to antiretroviral therapy (ART)

The association between patient characteristics and adherence to antiretroviral, based on viral load, is shown in Table 5. Findings revealed that 33.9 %(*n* = 62) of patients who have early ART initiation were more likely to be adherent than those patients (20.8 %, *n* = 38) who have delayed ART initiation from the date of HIV-confirmed diagnosis (*p* = 0.006). Meanwhile, 71.6 % (*n* = 131) of patients taking antiretroviral drugs, including two nucleoside reverse transcriptase inhibitors (NRTIs) and non-nucleoside reverse transcriptase inhibitors (NNRTIs), were more adherent (*p* = 0.011). Patient adherence was significantly associated with only two variables: the type of ART and timing of ART initiation.

### 3.6 Association between patient characteristics and success rate of antiretroviral therapy (ART)

Table 6 shows the association between patient characteristics and ART success based on virologic response after ART initiation. Most patients (32.8 %, n = 60) who had early ART initiation were more likely to have successful virologic from the date of HIV diagnosis (*p* < 0.05). This suggests that patients who started ART early after HIV detection and continued in the regimen will have improved viral suppression compared to patients who have delayed ART initiation. Furthermore, 55.4 % of the patients started ART within seven days of confirmed HIV diagnosis and had an improved virologic response; among those patients who started ART within seven days, 46.2 % (*n* = 60) had an improved virologic response and 44.6 % of patients initiated delayed ART (beyond 7 days of confirmed HIV diagnosis). Among the delayed initiators, 60.3 % initiated 8 days to 5 weeks of confirmed HIV diagnosis, the remaining 17.7 % started after 5 weeks, and 17.7 % to 29.3 % started delayed ART (after 2 months). Furthermore, the result of *x*^*2*^ tests of significance showed a significant association between ART initiation and virologic response, *x*^*2*^ (1,130) = 5.495, *p* = 0.019.

### 3.7 Strength of association between timing of ART initiation and viral suppression after taking ART

Based on the result, there is a significant association between the association between the date interval from the date of confirmed HIV diagnosis to the date of initial ART and virologic response after ART initiation, *η* = 0.683, *η*2 = 0.466 (Table 7). The eta-squared (*η*2) was calculated to determine the amount of variance in the virologic response attributed to the date intervals. Thus, the calculated Eta-squared is *η*2 = 0.466, which means that 46.6 % of the variance in virologic response after ART initiation can be attributed to date intervals from the date of confirmed HIV diagnosis to the date ART started; in other words, it is a strong effect, confirming the interpretation of the Eta coefficient test.

### 3.8 Association between patient adherence and success rate of antiretroviral therapy (ART)

Table 8 shows that there was a significant association (p < 0.05) between patient adherence and the success rate of ART, *x*^*2*^ (1, 183) = 167.956, *p* ≤0.001. As shown, 72.7 % (*n* = 133) of patients who tended to have good adherence had a successful virological response after taking antiretroviral drugs. In contrast, patients who were poorly adherent to antiretroviral drugs had unsuccessful virologic responses. These results suggest that patient adherence to ART and the success rate of ART are significantly associated. Patients with good adherence were most likely to show suppression of viral loads to below undetectable levels (<50 copies/mL), thereby decreasing the risk of transmitting the virus to others.

Adherence to ART has been defined by numerous authors, using various criteria. One of these criteria is viral suppression, a biological marker of adherence. Viral load monitoring is the most useful indicator of adherence and a routine component for monitoring individuals receiving ART [[15], [16], [17], [18]]. Measures of the success rate of ART include successful viral load suppression (<50 copies/mL), an increase in CD4+ count (>200 cells/μL), and the absence of AIDS-defining conditions after ART. In this study, we utilized viral load suppression to determine the association of the patient's demographic and clinical profiles with adherence and ART success rates.

## 4 Discussion

This study revealed that 73.8 % (n = 135) of treatment-naïve patients successfully suppressed the viral load to undetectable levels (<50 copies/ mL) after ART. Moreover, a high adherence rate in patients was associated with a reduction in viral load to undetectable levels (50 copies/ mL, *p* *≤*0.01). These findings support those of several studies showing that higher adherence rates are associated with higher levels of viral suppression [[18], [19], [20], [21], [22], [23], [24], [25], [26]].

This study also suggested that the virological response of patients after taking antiretroviral drugs showed favorable results in the suppression of viral loads. Several studies have also shown that routine viral load monitoring helps detect treatment failure earlier and improves the success of ART in terms of its preventive effect based on the model they developed, representing the course of individual viral load, immunological response, and survival in a cohort of HIV-infected patients receiving ART [[27], [28], [29]]. Viral load count serves as one of the bases for ensuring the long-term success of ART. Hence, these findings highlight viral load as a factor influencing the success rate of ART.

Furthermore, it was implied that the longer the interval of ART initiation from the date of confirmed HIV diagnosis, the more likely the patient was to have unsuccessful viral suppression and vice versa. This is also seen in several studies, which suggest that early ART initiation is associated with durable viral suppression, while late ART initiation is associated with virological failure, resistance, and sustained high viral load [[30], [31], [32], [33], [34], [35]]. Thus, the key to the long-term effectiveness and success of ART is associated with dealing with a high level of patient adherence. Poor ART adherence is a significant risk factor for the development of drug-resistant HIV strains, which can be transmitted to other individuals. Adherence to antiretroviral therapy has emerged as a key indicator for successful HIV treatment.

Although the retrospective study design employed in this study limits the ability to gather all relevant factors, the study still provides evidence supporting the importance of good adherence to antiretroviral therapy in treatment-naïve patients. This study highlights the importance of early initiation of antiretroviral therapy (ART) to improve treatment adherence and success rates among HIV-positive individuals in Davao City, Philippines. To enhance treatment outcomes, it is crucial to encourage the early initiation of ART. This approach can prevent disease progression, improve long-term health outcomes and reduce the risk of HIV transmission. Therefore, efforts are needed to promote early initiation of ART among HIV-positive individuals in Davao City.

## 5 Conclusions

These findings, gathered from a comprehensive ten-year retrospective cohort study, provided the critical importance of sustained ART adherence in effectively managing HIV/AIDS. This study provides valuable insights for healthcare providers and policymakers, highlighting the need for continued efforts to support and monitor patient adherence to therapy to improve treatment outcomes for HIV/AIDS patients in the Philippines.

## Ethical approval

The study was conducted in accordance with the Declaration of Helsinki and approved by the Institutional Review Board (or Ethics Committee) of The University of The Immaculate Conception.

## Author contributions

Jesille Mae Legarta, Reigner Jay Escartin, Rvin John Servillon, and Alfredo Hinay Jr. conceived the study. All authors managed data collection, analyses, and interpretation and critically reviewed the paper. Alfredo Hinay Jr. managed to acquire funding. All the authors have read and approved the final manuscript.

## CRediT authorship contribution statement

**Reigner Jay B. Escartin:** Writing – original draft, Methodology, Investigation, Formal analysis, Data curation, Conceptualization. **Rvin John T. Servillon:** Writing – review & editing, Writing – original draft, Validation, Supervision, Resources, Methodology, Investigation, Formal analysis, Data curation, Conceptualization. **Jesille Mae A. Legarta:** Writing – original draft, Validation, Software, Methodology, Investigation, Formal analysis, Data curation, Conceptualization. **Stephanie Kate G. Arriola:** Writing – original draft, Methodology, Investigation, Formal analysis. **Princess Faiqah A. Brahim:** Writing – original draft, Methodology, Investigation, Formal analysis. **Dennis Louis M. Braza:** Writing – original draft, Methodology, Investigation, Formal analysis. **Aireen V. Salva:** Writing – original draft, Methodology, Investigation, Formal analysis. **Alfredo A. Hinay:** Writing – review & editing, Writing – original draft, Visualization, Validation, Supervision, Software, Project administration, Methodology, Investigation, Funding acquisition, Formal analysis, Data curation, Conceptualization.

## Declaration of competing interest

The authors declare the following financial interests/personal relationships which may be considered as potential competing interests:

Alfredo A. Hinay, Jr. reports article publishing charges was provided by University of the Immaculate Conception. Alfredo A. Hinay, Jr. reports a relationship with University of the Immaculate Conception that includes: employment. If there are other authors, they declare that they have no known competing financial interests or personal relationships that could have appeared to influence the work reported in this paper.

## Data Availability

The datasets used and/or analyzed during the current study are available from the corresponding author upon reasonable request.
